# Treatment of Advanced Parkinson's Disease

**DOI:** 10.4061/2010/480260

**Published:** 2011-02-07

**Authors:** Sara Varanese, Zoe Birnbaum, Roger Rossi, Alessandro Di Rocco

**Affiliations:** ^1^New York University School of Medicine, Department of Neurology, Division of Movement Disorders, New York, NY 10016, USA; ^2^Robert Wood Johnson Medical School, Johnson Rehabilitation Institute, Edison, NJ 08818, USA

## Abstract

Patients at late stage Parkinson's disease (PD) develop several motor and nonmotor complications, which dramatically impair their quality of life. These complications include motor fluctuations, dyskinesia, unpredictable or absent response to medications, falls, dysautonomia, dementia, hallucinations, sleep disorders, depression, and psychosis. The therapeutic management should be driven by the attempt to create a balance between benefit and side effects of the pharmacological treatments available. Supportive care, including physical and rehabilitative interventions, speech therapy, occupational therapy, and nursing care, has a key role in the late stage of disease. In this review we discuss the several complications experienced by advance PD patients and their management. The importance of an integrative approach, including both pharmacological and supportive interventions, is emphasized.

## 1. Introduction

Advanced Parkinson's disease (PD), stage 4 or 5 of the Hoehn and Yahr Scale [1], is characterized by very limited mobility without assistance, severe motor deficits, risk of falls, and cognitive and psychotic problems. The mean time from disease onset to wheelchair-dependence is estimated at 14 years [2], although about a third of patients seem to have a relatively milder disease and remain stable for many years [3]. With the advent of the L-Dopa and other dopaminergic treatments, the progression of PD has become markedly slower. However, over the years treatment loses its efficacy, while a number of complications, such as motor fluctuations and dyskinesia, develop, probably due to the progressive loss of dopaminergic neurons and their striatal and cortical connections [4]. These complications are observed in 50% of patients after 5 years of disease and in 80% of patients after 10 years of treatment [5, 6]. However, the response to L-Dopa therapy predictability decreases over the years. 

 While worsening of motor function and the drug-induced motor complications represents a major challenge in patients with mid to advanced disease, in the advanced stage of PD the most troublesome and distressful complications are usually in the area of non-motor symptoms, including psychiatric and cognitive disorders, autonomic disturbances, and sleep disorders that significantly increase the need for supportive. These symptoms are frequently neglected in clinical practice due to limited consultation time, perception of the patient and caregivers that their symptoms are unrelated to the disease, or insufficient awareness of the clinicians who generally focus the consultation towards motor symptoms [7].

Psychosis and dementia are frequent and share a common pathophysiology in a significant proportion of patients [8, 9], where the impact on patients and family is variable. Dementia is associated with reduction in quality of life [10] and patient lifespan [11], psychosis is a risk factor for nursing home placement [12], and both are important sources of caregiver distress [13].

Management of motor and non-motor complications should be tailored to the individual patient. This implies a careful assessment of whether the symptom is a side effect of the medications or is related to the progression of the disease. In advanced disease, patients may also experience an enhanced sensitivity to small changes in L-Dopa or become more prone to adverse reactions to antiparkinsonian drugs. 

Proper supporting care becomes increasingly important in advanced PD. Rehabilitative and support services for patients and family also become key interventions as the disease reaches its more debilitating stages and pharmacological or surgical treatment becomes less relevant.

In this article we discuss the spectrum of the motor and non-motor complications seen in advanced stage PD and present an evidence-based review of current therapeutic options in the management of these complications.

## 2. Motor Disability

PD is defined as advanced when the patient is severely disabled. As per Hoehn and Yahr classification, patients in stage 4 are still able to walk and stand unassisted, but they are markedly incapacitated in their ability to perform activities of daily living (ADL). Patients in stage 5 are confined to bed or wheelchair unless aided.

Many patients in advanced stage range from stage 4 to 5 during the day because of the inconsistent and limited response to their medications.

Even when patients are still able to ambulate without assistance, limited motor ability due to marked bradykinesia and inability to perform fine and alternate movements lead them to dependency in ADLs, being unable to provide for basic personal care like dressing, bathing, and often feeding.

Advanced patients are frail individuals exposed to high risk of several unfavorable circumstances during daily activities, like falls.

The incidence of falls in advanced PD is high (40–70%) [14], even when patients are optimally medicated. Falls in advanced PD occur because of very unstable gait, loss of center of gravity, poor balance, orthostatic hypotension, side effects of medications like antidepressants and benzodiazepines, and disturbances of posture like camptocormia or retropulsion. Falls lead to injuries and fracture that further reduce patient independence and increase the risk of nursing home admission. Patients with previous falls often develop fear of falling which further limits their mobility, contributing to increased weakness and deterioration.

Because of the devastating consequences, an assessment of falls risk should be taken in all advanced PD patients. A combination of both disease-specific and balance- and mobility-related measures is necessary to accurately predict falls in patients with PD [15].

Treatment of falls implies a complex approach aimed at reducing all the potential risk factors, muscle strengthening, range of motion exercise and balance, and postural control training.

Although there is still insufficient evidence for effective prevention of falls, exercise interventions have shown to be effective at improving physical functioning, leg strength, balance, and walking [16]. Thus, physical interventions should be emphasized in advanced stages of disease, particularly as falls are currently not well addressed either by pharmacotherapy nor by subthalamic nucleus deep-brain stimulation (DBS) surgery.

The neuroanatomical substrates of posture and gait are poorly understood but a number of important observations suggest a major role for the pedunculopontine nucleus and adjacent areas in the brainstem. A recent double-blinded study reported a significant reduction in falls in the on and off medication states both at 3 and 12 months after pedunculopontine nucleus DBS as captured in the Unified Parkinson's Disease Rating Scale part II scores in six advanced Parkinson's disease patients with significant gait and postural abnormalities [17].

It has to be noted, however, that advanced patients are at high risk of short- and long-term complications from the DBS procedure, and surgical treatment is generally contraindicated in these patients. Furthermore, literature on pedunculopontine nucleus DBS is still limited, and long-term follow-up studies investigating safety and efficacy are unavailable.

## 3. Motor Complications

Long-term motor complications of PD are due to duration of disease and treatment, and to cumulative intake of L-Dopa, with several central and peripheral mechanisms involved. The progressive degeneration of the nigrostriatal dopaminergic transmission results in fewer and fewer terminals capable of taking up exogenously administered L-Dopa and converting it to dopamine for subsequent storage and release [6]. Unlike early and mid-stage PD patients advanced- and end-stage patients experience an enhanced sensitivity to small changes in plasma L-dopa levels [18, 19], that narrow the therapeutic window and negatively impact motor function. 

### 3.1. Wearing-Off, On-Off Fluctuations, and Management Strategies

“Wearing-off” refers to the recurrence of motor and non-motor symptoms preceding the scheduled dose of L-Dopa, while the on-off fluctuations are sudden unpredictable shifts between “well-” or “over-” treated status (on) and an undertreated state with severe Parkinsonism symptoms (off).

“Wearing-off” and on-off fluctuations overlap in advanced patients.

“Wearing-off” is a direct consequence of the nonphysiological, pulsatile dopaminergic stimulation, and its occurrence is generally predictable following the L-Dopa administration with progressive therapeutic window progressively narrowing over the years.

A plethora of sensory, psychiatric, and autonomic symptoms may be associated with the motor fluctuation. Patients, indeed, may present with paresthesia, pain, anxiety, shortness of breath, sweating, and other symptoms that may not be recognized as part of the L-Dopa response pattern [20].

Management strategy for “wearing-off” phenomena is focused on prolonging the effect of individual L-Dopa doses without increasing the pulsatile dopaminergic stimulation.

Strategies include fragmentation of dosing, with more frequent administration of lower doses, and use of COMT inhibitor (entacapone and tolcapone), MAO inhibitor (selegiline and rasagiline), and use of dopamine agonists.

Adjunctive therapy with a COMT inhibitor extends the duration of the L-Dopa effect, hence ameliorating wearing off, by blocking the COMT enzyme in the peripheral catabolism of L-Dopa. Potential adverse event, however, may arise from the COMT inhibitors. Increasing synaptic dopamine levels may also be associated with dyskinesia and increased L-Dopa toxicity leading to worsening of dementia and psychosis.

Fragmentation of oral therapy, with L-Dopa administered up to 6-7 times a day at about 3-hour intervals, is a commonly used and effective strategy [21]. However, lowering individual doses of L-Dopa may increase the risk of occasional drug failure or delayed response.

Substitution of regular with controlled-release L-dopa preparations may be particularly reasonable in end-stage patients [22], but the available extended release formulations are not always affective and reliable.

The use of dopamine-agonists (DAs), although theoretically useful in regulating fluctuations by direct stimulation of the postsynaptic receptors, is generally contraindicated in late-stage disease in order to avoid hallucinations and psychosis, and worsening of autonomic dysfunction.

The main challenge in controlling the on-off response is to improve the “on” time without increasing the dyskinesia.

In very late-stage PD this can be achieved using liquid formulations of L-Dopa [23], which can be prepared by dissolving ten 25/100 mg standard-release carbidopa/levodopa tablets and 2 g of ascorbic acid in 1 L of tap water [24].

Gastrointestinal dysfunction, with erratic gastric emptying worsening over the years, is a common cause of poor absorption of L-Dopa in PD. There is no gastric absorption of L-Dopa, indeed; so gastric emptying and transit via the pyloric sphincter are critical factors for regular intestinal absorption [25].

The liquid effervescent levodopa formulation of melevodopa (methyl-ester levodopa) plus carbidopa is a prodrug with a high solubility (about 250 times more than L-Dopa) in small volume of water, and it is able to reach quickly the small intestine where it is absorbed in a more regular and rapid way compared to solid formulations [26]. One clinical advantage of this formulation is that it avoids erratic absorption and the related unpredictability in the plasma L-Dopa concentration curve [27]. The drug is approved in certain European countries and currently under phase II investigation in the US.

Continuous infusion of levodopa/carbidopa gel through portable duodenal systems (Duodopa) using percutaneous endoscopic gastrostomy (PEG) can be a practical alternative [28, 29]. The infusion provides constant plasma levodopa concentration and continuous dopamine availability and receptor stimulation. This solution may be particularly reasonable in very advance patients with severe dysphagia, as the PEG may also be used for nutrition. Intrajejunal L-dopa/carbidopa gel infusion is effective in reducing off time, severity and duration of dyskinesia in advanced PD [30, 31]. Most importantly, a recent multicenter study demonstrated that intrajejunal L-dopa/carbidopa infusion provides a beneficial effect on several nonmotor complications, including cardiovascular, gastrointestinal, and urinary symptoms, sleep/fatigue, attention/memory, and pain [32]. Adverse event can occur, however, from the procedure or from the dislocation or occlusion of the intestinal tube. Advanced patients may also experience local complications at the site of entry, particularly inflammation and infections. 

Apomorphine subcutaneous infusion is also an effective option for patients with severe fluctuations poorly controlled by oral treatment [33]. Apomorphine infusion is often limited by the development of skin reaction at the site of injections after few years of treatment.

### 3.2. Dyskinesias

Dyskinesias are involuntary choreiform, twisting and turning movements invariably occurring in patients undergoing long-term L-Dopa treatment. Dyskinesias usually occur in “on” state, as chorea, myoclonus or dystonic movement. In end-stage patients dyskinesia may appear in off state as dystonic posture, especially in the lower limbs. Off dystonia is generally most troublesome upon morning awakening but in advanced disease may also develop complex twisting dystonic movements during the day. Because of the narrow therapeutic window at this stage it is also not uncommon for patients to experience diphasic dyskinesia. These are usually repetitive alternating movements occurring at the beginning as well as at the end of the interval between two L-Dopa doses [34].

Management of dyskinesias implies detailed understanding of the L-Dopa cycle.

The most common approach is to lower the single L-Dopa dose. Controlled-release levodopa may worsen dyskinesias, especially later in the day due to cumulative effect. Amantadine in doses between 100 mg and 400 mg can be effective, but side effects are frequent in more advanced patients and should be carefully monitored. These include edema, livedo reticularis, and confusional state or hallucinations and psychosis.

Clozapine, an atypical dopamine receptor antagonist, has been found to be effective in reducing dyskinesia in advanced patients [35, 36], and it may be particularly useful when hallucinations are also present. Advanced patients, however, are particularly prone to develop agranulocitosis, with high risks of infections, and thus the white cell count should be regularly monitored.

Recent evidence suggests that memantine is also effective in reducing dyskinesia when other options are contraindicated [37, 38].

Despite limited evidence-based data high-frequency subtalamic DBS (DBS-HFS) has been shown by several reports to be surgically safe and able to produce improvements in dopaminergic drug-sensitive symptoms and reductions in subsequent drug dose and dyskinesias are well documented. However, the procedure is associated with adverse effects, mainly neurocognitive, with side-effects created by spread of stimulation to surrounding structures, depending on the precise location of electrodes. The occurrence of cognitive complications limits the motor improvements induced by STN-HFS to a short period of time, because patients' quality of life is greatly impaired by the progressing cognitive disorder. In late stage of disease the rate of patients eligible for surgical treatment of PD is extremely low, due to age and general debilitation that significantly increase the risks of short- and long-term complications.

### 3.3. Drug Failure Response

As the disease progresses, the efficacy of L-Dopa progressively decreases and patients may not respond at all to administered doses. This phenomenon is more pronounced later during the day and may be related to poor gastric emptying and insufficient intestinal absorption. Domperidone is an effective option, where available. The neutral aromatic amino acids contained in dietary proteins may compete with L-Dopa for intestinal absorption and transport across the blood-brain barrier, thus limiting its efficacy and being responsible for the occurrence of motor fluctuations. Low-protein dietary regimens with protein redistribution by shifting protein intake to the evening are an effective strategy to ameliorate the response to L-Dopa. Low-protein products designed for chronic renal failure patients are also a safe, well-tolerated, and useful option for end-stage patients [39].

## 4. Nonmotor Complications

The neuroanatomical and neurochemical substrates of the majority of non-motor symptoms are still unclear, although the concept of Parkinson's disease as a six-stage pathological process introduced by Braak and collegues [40] provided critical information to understand the physiopathology of several nonmotor symptoms, such as sleep disorder, autonomic dysfunction, and visual hallucinations. 

Several studies have shown that non-motor symptoms impact significantly on quality of life and institutionalization is greater than for the motor symptoms [41, 42]; so in recent year attention was focused on the development of measures specifically designed to recognize and quantify these symptoms in advanced patients, and they are now also widely used in the clinical trials.

The development of clinical measures useful in recognizing and quantifying these symptoms deeply improved the clinical care as well as the clinical trials.

The Non-Motor Symptoms Scale, for instance, is a 30-item scale for assessment of nine dimensions (cardiovascular, sleep/fatigue, mood/cognition, perceptual problems, attention/memory, gastrointestinal, urinary, sexual function, and miscellany), that has proven to be a valid, reproducible, and accurate tool in rating severity and frequency of non-motor symptoms in PD [43, 44].

### 4.1. Dementia

Community-based studies of dementia in patients with PD have reported a prevalence between 28% and 44%, with longitudinal studies estimating that dementia occurs in up to 75% of patients [45]. The pattern of deficits is similar to dementia with Lewy bodies and differs from that in Alzheimer's disease for the predominant involvement of executive, visuospatial, and attention dysfunction and for the presence of cognitive fluctuations [46–49].

The cognitive symptoms are a consequence of dopaminergic depletion [50] in the corticostriatal loop and of dysfunction of the cholinergic system [51]. Serotoninergic and noradrenergic mechanisms may also be involved, though their role is not well defined.

Dopaminergic replacement does not lead to cognitive improvement or may even worsen it, but cholinergic enhancement can instead be helpful. Cholinesterase inhibitors, in fact, may be effective in ameliorating cognition, but their tolerability seems variable due to peripheral cholinergic adverse effects and in some case can worsen motor functions. Rivastigmine seems the most useful agent [52], while more controversial is the benefit produced by donepezil [53, 54].

Avoiding the medications that can possibly worsen dementia, like anticholinergics and DA-agonists, as well as maintaining L-Dopa at the lowest effective doses, is certainly a key strategy to contain confusion, hallucinations, and psychosis in advanced patients [55].

### 4.2. Hallucinations and Psychosis

Behavioral disorders, and especially hallucinations, illusions, and other psychotic symptoms, are also frequent in advanced PD with frequency rates ranging from 25 to 30%. Resembling very closely those seen in dementia with Lewy bodies, psychotic symptoms in PD are represented by delusions (false and fixed beliefs maintained despite evidence to the contrary) and, particular, hallucinations (abnormal perceptions that can involve any sensory modality in the absence of a physical stimulus). Visual hallucinations, simple or complex in form, are the most common psychotic symptom in advanced PD patients, typically occurring in dim surrounding, but often occurring through the entire day in late-stage patients [56].

A range of factors contributes to the development of hallucinations and psychosis in PD, including intrinsic pathology and dopaminergic replacement therapy.

In the treatment of these complications the first step should always be to evaluate the role of drugs that can potentially induce or worsen psychosis, such as amantadine, anticholinergics, COMT-inhibitors, and DA-agonists. These drugs should be tapered off, balancing the effect on psychosis with worsening of motor function.

All precipitating events, like urinary and pulmonary infections, cerebrovascular events, and metabolic dysfunctions, should be also carefully investigated and treated if possible, as even mild metabolic imbalance or infection can profoundly affect the development of psychotic symptoms.

Decreasing the dose of L-Dopa should also be considered when severe psychosis persists, even though this action could worsen parkinsonim.

All traditional antipsychotic drugs, such as haloperidol, aripriprazole, and chlorpromazine, should be avoided because of the high sensitivity of PD patients to the motor adverse effects induced through potent antagonisms of D_2 _receptors.

Clozapine and quetiapine are the only two newest antipsychotic that should be considered atypical, thus safe in PD, due to their predominant affinity for D_1_ and D_4 _receptor and low affinity for D_2_ receptors.

There is a wealth of evidence demonstrating the efficacy and tolerability of clozapine in PD, but its use is limited by the need of weekly blood testing for the initial 6 months of treatment [57]. A more practical alternative is represented by quetiapine. Unlike clozapine, quetiapine does not require monitoring of blood cell counts and it is effective in suppressing hallucinations and psychosis in the majority of patients at relatively low doses, ranging from 12.5 mg to 100 mg.

Main side effects of quetiapine and clozapine are sedation and postural hypotension.

### 4.3. Depression and Anxiety

Depression affects 40–60% of patients with PD and appears to be a major determinant of health-related quality of life in PD [58].

In some cases depression occurs during off periods; thus controlling the on-off fluctuation can improve depression.

Sedating antidepressants, like tricyclic (TCA), and more activating antidepressants, like selective serotonin reuptake inhibitors (SSRIs) and serotonin-norepinephrine reuptake inhibitors (SNRIs), are useful but significantly limited in advanced patients by the ancticholinergic and orthostatic negative effects. SSRIs are also contraindicated in patients receiving slelegiline, because of the potential drug-drug interation leading to “serotonin syndrome”.

S-Adenosyl-methionine (SAMe), a natural molecule present in all eucaryotic cells that participates as methyl group donor to a number of metabolic events, is reported to have an effective antidepressant effects [59], without worsening of Parkinsonism [60].

Anxiety often occurs during “off” periods and improves with better control of motor symptoms but can be a major source of distress for patients even during “on” state. Low doses of benzodiazepines are effective when anxiety is persistent and debilitating but may cause amnesia and confusion in advanced patients and are a risk factor for falls.

### 4.4. Sleep Disorders

Sleep disorders occur in almost all patients with advanced PD, and they consist of sleep fragmentation, REM sleep behavior disorders (RBDs), excessive daytime sleepiness, and altered sleep-wake cycle.

Sleep fragmentation can be caused by difficulty turning in bed or nocturnal dystonia and can be ameliorated with controlled-release levodopa. Increased nocturnal urinary frequency can also affect sleep and can be controlled by reducing the amount of liquids in the evening, when anticholinergic drugs are contraindicated.

RBD is a disruption of the normal REM sleep cycle, in which the paralysis that normally occurs during REM sleep is incomplete or absent, making the patient “act out” their dreams, that usually are vivid, intense, and violent. Dream-enacting behaviors can be complex, including talking, yelling, punching, kicking, jumping from bed, and grabbing, with great distress for the patient and bed partner. RBD also prevents physiological nocturnal restoration of dopamine reseverve in cells, with worsening of parkinsonian symptoms. RBD improves when dopaminergic medications are reduced at bedtime. When RBD persists, low doses of clonazepam are effective and should be considered.

Modafinil, a wake promoting agent approved for narcolepsy, is effective in ameliorating daytime sleepiness induced by dopamine-agonists without significant side effects [61] and can be helpful in ameliorating alertness in advanced PD.

### 4.5. Autonomic Dysfunction

#### 4.5.1. Orthostatic Hypothension (OH)

OH is defined as a fall in systolic blood pressure below 20 mmHg and I diastolic pressure below 10 mmHg within 3 minutes of standing. Orthostatic intolerance related to OH results from a reduction of cerebral perfusion when upright and presents in severe cases with lightheadedness or syncope, exposing the patient to high risk of fall.

Careful education of patients and caregivers on factors that can trigger the OH symptoms, like avoiding rapid changes of position or straining during micturition or defecation, is essential in the management of OH.

Fluid intake, particularly in the morning, should be maintained at around 2 L of water daily and at least 8 g of sodium chloride is recommended to ensure adequate hydration [62].

Antihypertensive therapy, when present, should be reconsidered and eventually discontinued. Thromboembolic elastic stocking and abdominal binders can be helpful and should be encouraged.

When OH becomes more severe, it is necessary to start pharamchological agents such as plasma volume expander, like fludrocortison, and vasoactive agents, like midodrine.

#### 4.5.2. Dysphagia, Nutrition, and Hydration

Severe dysphagia occurs frequently at late stage of disease causing weight loss, malnutrition, dehydration, and significantly increasing the risk of inducing aspiration pneumonia and death. 

In order to make the swallow safer and more effective swallowing maneuvers, like the supraglottic swallow maneuver, the super supraglottic swallow maneuver, the Mendelsohn maneuver, and the effortful swallow maneuver, should be taught to patients.

Dysphagia for fluid can be controlled adding thickening agents, or thickeners, to liquids, increasing their viscosity without substantially modifying their other properties, such as taste. They provide body, increase stability, and improve suspension of added ingredients. Some thickening agents are gelling agents, forming a gel that can be swallowed by patients significantly reducing the risk of chocking.

When dysphagia becomes more severe, PEG should be considered. In this phase PEG could be a useful solution to guarantee to patients' adequate food and fluid intake as well as dopaminergic therapy through infusion.

#### 4.5.3. Genitourinary and Elimination

Constipation is a common and early manifestation of PD but in late stage can become particularly severe due to the combination of anti-PD medications, slowed intestinal motility, immobility, and dehydration. Constipation should be well managed in order to avoid bowel occlusion and in order to ensure proper absorption of L-dopa and other medications. Dietary supplementation of fibers that stimulate intestinal motility should be encouraged as well as increased fluid intake. A conservative therapeutic option is administration of macrogol (polyethylene glycol), which can lead to marked improvement [63]. 

Many late-stage PD patients face urinary problems such as urgency or frequency or stress incontinence, which can cause anxiety and feelings of social isolation. Overactive bladder is the result of loss of normal inhibition by the basal ganglia and the frontal cortex to the sacral spinal cord [64]. Anticholinergics are commonly used to inhibit the overactive bladder, although their use should be discouraged in late-stage patients due to cognitive and other central anticholinergic adverse effects [65]. Newer generation of peripheral anticholinergics, like trospium, is better tolerated and can be used sometimes even in advanced patients. Recently, botulinum toxin injections in the detrusor muscle have demonstrated marked efficacy in reducing the urinary frequency with no side effects [66]. 

Reduced mobility and difficulty toileting often lead to the use of urinary pads or catheters at end stage of disease, exposing the patients to high risk of urinary dangerous infections when hygienic measures are not appropriate.

## 5. Supportive Care

Supportive care in advanced PD patients should include physical and rehabilitative therapy, occupational therapy, speech therapy, social work, and nursing care. These care services could greatly benefit late-stage patients by prolonging independency in the ADL and reducing complications like pain, decubiti, and falls.

### 5.1. Mobility

Full mobility should be encouraged and maintained as long as possible. Occupational and physical therapy should be encouraged whenever possible. Individual rehabilitative therapy sessions should be encouraged two to three times weekly for 30- to 40-minute durations even at late-stage when the patient is able to safely ambulate. Falls are perhaps the greatest concern for late stage PD patients who are still mobile, and patients should be discouraged to stand or walk without assistance at very late stage of disease. If patients are bedridden, residual mobility should be maintained through active and passive movement exercises, frequent position changes, and breathing exercises to prevent complications associated with being bedridden, such as decubitus, contracture, pain, and pneumonia [67].

### 5.2. Nutrition, Hydration, and Genitourinary Care

Malnutrition is a common problem in advanced PD patients. It is caused by difficulty feeding, altered satiety mechanism, diminished gastric and intestinal motility, inactivity, lack of appetite, dysphagia, and metabolic syndrome. In patients still able to eat independently, meal and portion sizes should be monitored in order to provide sufficient nutrition. Any effort, including compensatory strategies, should be considered to delay the PEG placement. Adequate hydration is another concern for late-stage PD patients, since even mild temperature change can lead to relative dehydration and exacerbate confusion and OH and cause syncope. Many patients become embarrassed when eating or drinking, and nursing assistance, can assure adequate nutrition and hydration through a nonjudgmental caregiver that assist patients with the administration of meals.

### 5.3. Communication

Difficulty with speech with severe dysarthria, hypophonia, tachylalia, and freezing of speech is another problem associated with late-stage PD and leads to significant source of frustration for patients and families. Speech therapy should be encouraged whenever possible. The Lee Silverman Voice treatment has been shown, clinically and scientifically, to be a powerful method of improving speech and related functions such as swallowing and facial expression in PD, with documented Improvement in vocal loudness, voice quality, prosody, and speech articulation, sustained at 1-year and 2-year follow-ups [68]. Simplified and codified communications (like asking yes/no questions, or by using alphabet boards or speaking dictionaries) can become the only way of effective communication [69] and should be considered.

## 6. End of Life Care

When patients with advanced PD encounter a medical illness requiring an extended rehabilitation stay, they are often transferred to subacute rehabilitation facilities with no expertise in treating Parkinson's disease. These transfers often lead to an inevitable decline due to worsening of dementia, psychosis, and social withdrawal. Nursing home placement should be delayed as long as possible, because of the well-known risk of reduced survival. As death approaches for late-stage PD patients, it is important to provide them with the best care possible in a passionate environment. Many patients choose to do this through hospice care. Support to families, through social work and psychological counseling, should be offered at this time.

## 7. Conclusion

The management of end-stage PD challenges clinicians, patients, and families in many ways.

The main goal should be to maintain acceptable levels of functioning through careful balance not limited to drug management, but including strong and supportive services. 

Many patients with advanced PD, in fact, benefit from a more intensive intervention to address the complexity of the disease. Medication management can become arduous with on/off fluctuations and dyskinesias, frequent falls, constipation, blood pressure instability, cardiac problems, and other medical complications of PD developing and becoming more severe as the disease progresses. The process is further complicated when speech is affected, and swallowing becomes difficult with malnutrition and risk of developing aspiration pneumonia. Psychological problems often accompany these later stages of the disease, including anxiety, depression, and insomnia. Cognitive problems and hallucinations also are prominent. 

There comes a time when it becomes too difficult to manage all these complexities at home. Patients and caregivers become overwhelmed, often with unnecessary catastrophic consequences. Institutionalization typically follows the dramatic period of declining health and diminished ability to cope. For most persons with advanced PD the quality and dignity of a life at home are much superior to what they can ever expect in a nursing home. A well-designed interdisciplinary intervention can, in most cases, resolve many problems and render the care of patients much more manageable at home. Unfortunately, medical facilities are unprepared to accommodate the needs of the neurologically frail and complex PD patients.
